# Prediction of cardiovascular events after carotid endarterectomy using pathological images and clinical data

**DOI:** 10.1007/s11548-024-03286-w

**Published:** 2024-11-09

**Authors:** Shuya Ishida, Kento Morita, Kinta Hatakeyama, Nice Ren, Shogo Watanabe, Syoji Kobashi, Koji Iihara, Tetsushi Wakabayashi

**Affiliations:** 1 Graduate School of Engineering, Mie University, 1577, Kurimamachiya-Cho, Tsu, Mie 514-8507 Japan; 2 National Cerebral and Cardiovascular Center, 6-1, Kishibe Shimmachi, Suita, Osaka 564-8565 Japan; 3 Graduate School of Engineering, University of Hyogo, 2167, Shosha, Himeji, Hyogo 671-2280 Japan

**Keywords:** Multimodal learning, Anomaly detection, Pathological image, Outcome prediction, Cardiovascular events

## Abstract

**Purpose:**

Carotid endarterectomy (CEA) is a surgical treatment for carotid artery stenosis. After CEA, some patients experience cardiovascular events (myocardial infarction, stroke, etc.); however, the prognostic factor has yet to be revealed. Therefore, this study explores the predictive factors in pathological images and predicts cardiovascular events within one year after CEA using pathological images of carotid plaques and patients’ clinical data.

**Method:**

This paper proposes a two-step method to predict the prognosis of CEA patients. The proposed method first computes the pathological risk score using an anomaly detection model trained using pathological images of patients without cardiovascular events. By concatenating the obtained image-based risk score with a patient’s clinical data, a statistical machine learning-based classifier predicts the patient’s prognosis.

**Results:**

We evaluate the proposed method on a dataset containing 120 patients without cardiovascular events and 21 patients with events. The combination of autoencoder as the anomaly detection model and XGBoost as the classification model obtained the best results: area under the receiver operating characteristic curve, accuracy, sensitivity, specificity, and F1-score were 81.9%, 84.1%, 79.1%, 86.3%, and 76.6%, respectively. These values were superior to those obtained using pathological images or clinical data alone.

**Conclusion:**

We showed the feasibility of predicting CEA patient’s long-term prognosis using pathological images and clinical data. Our results revealed some histopathological features related to cardiovascular events: plaque hemorrhage (thrombus), lymphocytic infiltration, and hemosiderin deposition, which will contribute to developing preventive treatment methods for plaque development and progression.

## Introduction

Myocardial infarction and stroke are leading causes of death as severe complications of the circulatory system [1, 2], and various studies have been conducted to elucidate their pathogenesis and establish preventive and therapeutic methods [3, 4]. Although many prognostic factors have been reported for the occurrence of cardiovascular disease [5–8], practical strategies for the prevention and treatment of cardiovascular events have not been established yet [9].

Carotid endarterectomy (CEA) is performed to treat carotid artery stenosis, in which the carotid artery becomes narrowed or blocked due to the buildup of fatty deposits (plaque). Since the carotid artery is a primary passageway for blood to the brain, the formation of plaques can reduce cerebral blood flow or cause plaques to rupture, resulting in stroke. There are two types of plaques: stable plaques with high fiber content and unstable plaques with a high-fat content that may cause cerebral infarction. CEA is a procedure to remove these plaques to improve blood flow. Several reports support CEA for patients with intense plaque stenosis [10–13], in which CEA reduces the incidence of cerebral infarction, preventing primary stroke in more than 60% of cases and secondary stroke in more than 50% of cases [13]. However, asymptomatic stroke occurs in up to 34% of patients after CEA [14], and the 5-year risk of stroke is as high as 6.9% [15], thus it remains difficult to predict a patient experiences the long-term complication after CEA [5, 6]. Recent studies reported the carotid plaque score (CPS), evaluating atherosclerosis in the carotid artery based on the number and the size of plaques obtained using ultrasound echo images, is an essential index in predicting ischemic stroke and major adverse cardiovascular events [16–19]. The index has the possibility to predict the incidence of postoperative cardiovascular disease [17–19], which suggests pathological images of carotid artery plaques have the potential to predict the patient’s prognosis.

There are several studies attempted to obtain a surrogate marker of cardiovascular diseases: carotid intima-media thickness (IMT) was measured in ultrasound echo images to examine its relation to the progression of cardiovascular disease [3, 4], the carotid atherosclerosis scores from magnetic resonance (MR) images to predict plaque progression [20], and clinical data to predict prognosis after CEA surgery [5, 6, 21–23]. However, the pathological images of carotid plaques were not considered to explore the indicator of predicting systemic atherosclerotic lesions and the development of new cerebral infarctions or other cardiovascular diseases. The in-depth analysis of pathological images can potentially reveal unknown factors that cause these symptoms. This research explores pathological or clinical prognostic factors to reduce cardiovascular events after CEA, which will also contribute to developing preventive methods for plaque development.

In a related study, Mobadasany et al. [24] predicted prognosis using pathological images. They proposed a method to predict survival time from pathological images of brain tumor patients using a model named survival convolutional neural networks (SCNN), which combines VGG19 and the Cox proportional hazards model. The experiment was performed on 769 patients with brain tumors, using 1061 pathological images, achieving a C-index, which is an agreement of predicted survival time and actual survival time, of 0.741 for the SCNN model. Foersch et al. [25] predicted leiomyosarcoma patients’ survival and death at two years after the acquisition of pathological images using Densenet121. Experiments using 139 pathological images achieved an area under the receiver operating characteristic curve (ROC-AUC) of 0.91. Given that prognosis can be predicted from pathological images of brain tumors and leiomyosarcoma, we hypothesize that prognosis can also be predicted from pathological images of carotid arteries.

Matsuo et al. [21] employed 17 types of clinical data, such as patient age, pre-treatment modified Rankin scale (mRS), and the presence of diabetes, to predict whether ischemic stroke occurred within 30 days after CEA and carotid artery stenting (CAS) procedures. They compared five machine learning (ML) models, and XGBoost achieved the best results: area under the receiver operating characteristic curve (ROC-AUC) of 0.719 and an accuracy of 70.8%. This performance was comparable to that of the surgeon. Li et al. also conducted a study using clinical data to predict the incidence of cardiovascular disease and survival within 30 days [22] and one year [23] after CEA surgery, and the XGBoost model showed the best results in both cases, with ROC-AUC exceeding 0.90.

Recent studies have also shown the potential of anomaly detection in medical images such as X-ray images, MRI images, and pathological images [26–30]. In a study by Siddalingappa et al. [28], anomaly detection was used to estimate benign and malignant lung cancer from CT images. The model used a convolutional autoencoder, which was trained using only CT images of benign patients. They defined the anomaly score by the mean squared error (MSE) between the input image and the output image of the model and achieved an ROC-AUC of 0.972 on the test set.

This paper employs pathological images and clinical data to build a predictive method for cardiovascular events within one year after CEA to clarify the histopathology and carotid plaque morphology associated with the repeated event. The proposed method first extracts patches from pathological images of carotid plaques. Then, the anomaly detection model evaluates the abnormality of the tissue and calculates the pathological risk score for cardiovascular events. Finally, the classification model predicts the future onset of cardiovascular events from the pathological risk score and clinical data.

## Dataset

This study obtained pathological images and clinical data from 141 patients who underwent CEA at the National Cerebral and Cardiovascular Center between May 9, 2002, and September 1, 2010. We use hematoxylin and eosin (H&E) stained specimens, that a plaque resected by CEA is fixed with formalin, paraffin-embedded, cut into very thin slices, and finally the digital slide scanner acquires its image as the whole slide imaging (WSI). The images were taken at 40 × magnification using the NanoZoomer S210 digital slide scanner (Hamamatsu Photonics K.K.). Pathologists performed a pathological evaluation, and the outcome was defined as the composite of stroke, including asymptomatic diffusion-weighted imaging (DWI) hyperintense lesions and myocardial infarction within one year after CEA. The 120 patients who did not have a stroke or myocardial infarction were defined as normal data, and the 21 patients who had a cardiovascular event were defined as abnormal data (Table 1). A total of 18 parameters were used as clinical data of the patients (Table 2). Although the proportion of males is about 94% (132 males and 9 females), the prevalence of carotid artery stenosis is overwhelmingly higher in males in Japan [21, 31]. Missing values were filled in by averaging. This study was approved by the Ethics Committee of the National Cerebral and Cardiovascular Center, and informed consent was obtained from the patients.

**Table 1:** Number of subjects used in the experiment

Label	Event (1 year)	# of patients	# of pathological images
Normal	No	120	197
Abnormal	DWI high signal	10	16
	Myocardial infarction	7	11
	Stroke	4	4

**Table 2:** Patient’s clinical data

Variable		Normal (*n* = 120)	Abnormal (*n* = 21)
Characteristics	Age, mean (SD)	70.1 (6.5)	69.5 (6.7)
	Male sex, *n* (%)	111 (92.5)	21 (100)
	Vessel stenosis rate, mean (SD)	68.5 (26.4)	68.7 (27.0)
	NIHSS (national institute of health stroke scale), mean (SD)	1.2 (2.68)	3.0 (5.7)
	MPRAGE (magnetization prepared rapid acquisition with gradient echo) [32], mean (SD)	1.9 (1.0)	2.1 (1.2)
	Smoking history, *n* (%)	72 (60.0)	12 (57.1)
Past history/comorbidity	Hypertension, *n* (%)	96 (80.0)	19 (90.4)
	Diabetes mellitus	32 (43.3)	4 (19.0)
	Dyslipidemia, *n* (%)	86 (71.6)	18 (85.7)
	Arteriosclerosis obliterans, *n* (%)	16 (13.3)	1 (4.7)
	Coronary artery disease, *n* (%)	40 (33.3)	5 (23.8)
Medications	Aspirin, *n* (%)	108 (90.0)	17 (80.9)
	Ticlopidine, *n* (%)	21 (17.5)	7 (33.3)
	Clopidogrel, *n* (%)	19 (15.8)	3 (14.2)
	Cilostazol, *n* (%)	19 (15.8)	3 (14.2)
	Warfarin, *n* (%)	0 (0)	8 (38.0)
	Heparin/argatroban, *n* (%)	14 (11.6)	4 (19.0)
	Statins, *n* (%)	80 (66.6)	13 (61.9)

## Methods

### Overview

**Fig. 1:**
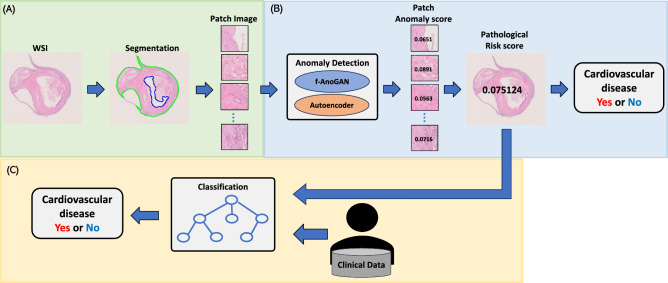
Overview of the proposed method. **A** Determines the contours of pathological tissues using Otsu’s algorithm, and small patch images are extracted from the pathological tissue region. **B** The pathological risk score of a WSI is computed from patch-level anomaly scores obtained by the anomaly detection model. **C** The statistical machine learning-based classification using the pathological risk score and clinical data attempts to improve the prediction accuracy

Figure 1 illustrates the flowchart of the proposed method. Step (A) determines the contours of pathological tissues using Otsu’s algorithm to extract small patch images from the pathological tissue region. In Step (B), the pathological risk score of a WSI is computed from patch-level anomaly scores obtained by the anomaly detection model (f-AnoGAN or autoencoder). Although the thresholding to the pathological risk score would predict the future onset of cardiovascular diseases, the statistical machine learning-based classification using the pathological risk score and clinical data attempts to improve the prediction accuracy in Step (C).

#### Preprocessing

Pathological images have a large number of pixels to feed into a well-known CNN model; therefore, it is divided into small patches to train a CNN [24, 25, 29, 30]. The preprocessing of WSI utilized a part of CLAM [33] implementation. Initially, the image was down-sampled 32 × using OpenSlide, followed by conversion from RGB to HSV color space. After applying a median filter, Otsu’s algorithm was used for binarization. Additional morphological closing was applied to fill small gaps and holes in the tissue, thereby segmenting the tissue regions from the WSI. From the segmented tissue regions, a 256 × 256 pixels stride sliding window is used to extract 256 × 256 pixels patch images. The flow of tissue region extraction and patch image creation is shown (Fig. 1A). The area surrounded by the green line is the tissue area to be extracted, and the area surrounded by the blue line is the hole, which is not extracted.

#### Anomaly detection

Anomaly detection, which identifies features extracted from distributions that differ from the distribution of features in the training data, is applied to locate anomalous areas in pathological images and to calculate a pathological risk score for cardiovascular events (Fig. 1B). Here, we apply anomaly detection to pathological images to detect abnormal pathology that does not frequently appear in normal patients, aiming to identify pathological factors associated with cardiovascular events to estimate the long-term prognosis.

This study compares unsupervised models widely used for anomaly detection: f-AnoGAN [34] and convolutional autoencoder [35]. Both models are trained on normal images only. At inference, those models calculate the anomaly score by comparing the input and generated images. After calculating the anomaly score for a patch image $\text{A}\left(x\right)$, the pathological risk score ${A}_{p}$ of a patient is calculated by Eq. (1).

1 $${A}_{p}=\frac{{\sum }_{x\in \text{X}}A\left(x\right)}{\left|\mathbf{X}\right|}$$

where $\mathbf{X}$ is the patch group for which the anomaly score is calculated, and the average of the anomaly scores for the number of patches per patient is obtained.

##### f-AnoGAN

f-AnoGAN [34] is a GAN for anomaly detection and is a faster version of AnoGAN [36]. f-AnoGAN aims to speed up inference by eliminating gradient descent search during inference. Three architectures have been introduced for f-AnoGAN, and the izif architecture is adopted in this study. The WGAN-GP (Wasserstein GAN with Gradient Penalty) [37] and StyleGAN2 [38] are used as the GAN model. The anomaly score $A\left(x\right)$ for each batch of input images in f-AnoGAN is calculated by Eq. (2). This equation calculates the sum of the squared difference between the input image $x$ and the generated image $G(E(x))$, and the squared difference between the feature vectors in the discriminator $f\left(\cdot \right)$;

2 $$A\left(x\right)=\frac{1}{n}{\left|x-G\left(E\left(x\right)\right)\right|}^{2}+\frac{1}{{n}_{d}}{\left|f(x) - f(G(E(x))\right|}^{2}$$

where $n$ is the total number of pixels in input image $x$, ${n}_{d}$ is the number of feature dimensions extracted by $f\left(\cdot \right)$. $G(E(x))$ is the generated image for the input image $x$, where $E\left(\cdot \right)$ is the encoder output and $G\left(\cdot \right)$ is the decoder output. The feature representation $f\left(\cdot \right)$ is obtained from the layer before the final layer of the discriminator.

##### Autoencoder

The encoder part consists of three convolutional layers, each followed by a ReLU activation function and max pooling. Down-sampling results in a 128-channel feature map, which is then flattened and converted to a 512-dimensional intermediate representation. It is then inversely transformed to the original feature map size and passed to the decoder part. The decoder part contains three inverse convolution layers: first two layers followed by ReLU and final layer followed by a sigmoid activation function. The anomaly score $A\left(x\right)$ for each batch of images in autoencoder is given by the mean squared error (MSE) of the input and the generated image;

3 $$A\left(x\right)=\frac{1}{n}{\left|x-D\left(E\left(x\right)\right)\right|}^{2}$$

where $n$ is the total number of pixels in input image $x$. Since $E\left(\cdot \right)$ is the encoder output and $D\left(\cdot \right)$ is the decoder output, $D\left(E\left(x\right)\right)$ is the reconstructed image for the input image $x$.

#### Classification

After calculating the pathological risk score by the anomaly detection model, 18 types of clinical data were combined to perform the binary classification to predict the cardiovascular events from 19 features (Fig. 1C). We compared with two classification models in the experiment: XGBoost and LightGBM.

## Experiments and results

### Experimental setup

For the anomaly detection models, f-AnoGAN and autoencoder, the proposed method uses pathological images of 121 WSIs of 70 normal patients for training and the remaining 76 WSIs of 50 normal patients, and 31 WSIs of 21 abnormal patients for calculating pathological risk scores. The training settings for f-AnoGAN (WGAN-GP) are the following: the number of epochs is 300, the batch size is 64, the learning rate is 0.00005, the optimization function is RMSprop, and the number of dimensions of latent variables is 1024. The training settings for f-AnoGAN (StyleGAN2) are the following: the number of iterations is 10,000, the batch size is 16, the learning rate is 0.002, and the number of dimensions of latent variables is 512. The training settings for autoencoder are as follows: the number of epochs is 300, the batch size is 64, the learning rate is 0.001, and the optimization function is Adam. We chose the trained GAN model with the lowest Fréchet inception distance (FID) score on the training set. The other hyperparameters followed the official implementation. The early stopping terminated the encoder training after the MSE loss did not improve for 30 consecutive epochs.

The experimental data for the training, validation, and testing of the cardiovascular events prediction model using pathological risk score and clinical data consist of 71 patients comprising 50 normal and 21 abnormal patients who were not used in the training of anomaly detection model. The random sampling splits these 71 patients into training and test sets in a 7:3 ratio, ensuring that all data from a patient were assigned to the same subset. To avoid the hyperparameter optimization on test sets, a five-fold cross-validation and grid search were performed in the training set to obtain the optimal prediction model, achieving the lowest log loss. The trained prediction model performs the cardiovascular events prediction for the test set to validate its prediction performance. This process was repeated 100 times with different combinations of subjects to evaluate the prediction performance using the average value of these repetitions. In this classification experiment, we employed the Youden index to determine the threshold to distinguish the events-onset subjects from normal subjects.

### Performance evaluation of cardiovascular event prediction

Table 3 shows the results of cardiovascular event prediction. The methods combining pathological risk score and clinical data (methods #3 to #8) achieved better prediction performance than methods only using clinical data (methods #1 and #2). The autoencoder and XGBoost (method #7) achieved the best performance except for sensitivity, with ROC-AUC of 0.819 and F1-score of 0.766.

**Table 3:** Prediction results of cardiovascular events combining pathological risk score and clinical data. Methods #1 and #2 used only clinical data, while methods #3 ~ #8 used pathological risk score calculated by using three different types of anomaly detection models. XGBoost or LightGBM performs the cardiovascular events prediction for each condition of pathological risk score computation. The evaluation index ROC-AUC, accuracy, and F1-score are shown with 95% confidence intervals

#	Pathological risk score	Classifier	ROC-AUC (95% CI)	Accuracy (95% CI)	Sensitivity	Specificity	F1-score (95% CI)
1	–	XGBoost	0.691 (0.672–0.711)	0.699 (0.680–0.717)	0.831	0.637	0.637 (0.620–0.653)
2		LightGBM	0.701 (0.679–0.724)	0.695 (0.676–0.714)	0.841	0.627	0.640 (0.624–0.657)
3	f-AnoGAN (WGAN-GP)	XGBoost	0.772 (0.755–0.788)	0.780 (0.762–0.798)	0.814	0.764	0.708 (0.692–0.724)
4		LightGBM	0.779 (0.760–0.797)	0.787 (0.770–0.804)	0.794	0.783	0.706 (0.688–0.724)
5	f-AnoGAN (StyleGAN2)	XGBoost	0.728 (0.708–0.749)	0.730 (0.710–0.749)	**0.843**	0.677	0.670 (0.653–0.687)
6		LightGBM	0.726 (0.706–0.746)	0.723 (0.703–0.743)	0.828	0.674	0.659 (0.641–0.678)
7	**Autoencoder**	**XGBoost**	**0.819 (0.802–0.836)**	**0.841 (0.823–0.858)**	0.791	**0.863**	**0.766 (0.747–0.784)**
8		LightGBM	0.810 (0.791–0.829)	0.817 (0.799–0.835)	0.821	0.815	0.747 (0.728–0.765)

The significance of bold best overall model for Pathlogical risk score, Classifier, and Model are in bold, and the highest value for each evaluation index is bold

### Comparison of pathological risk score calculation models

**Fig. 2:**
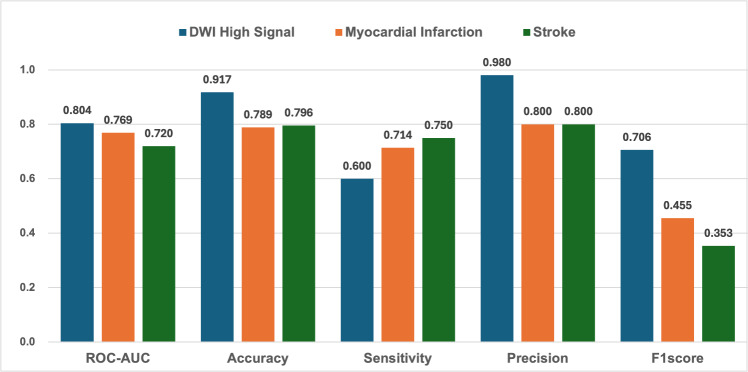
The performance for each disease using pathological risk scores for 71 patients obtained in the autoencoder model: DWI high signal, myocardial infarction, stroke

As shown in Table 3, the pathological risk score improved the classification performance. For further analysis of the pathological risk score calculated using three anomaly detection models, we conducted binary classification experiments on 71 patients using the Youden index threshold of pathological risk scores in Table 4. Since autoencoder achieved the best ROC-AUC and F1-score in the comparison, the disease-wise prediction using autoencoder for three diseases was conducted as shown in Fig. 2. Note that the disease-wise experiment cannot employ common patients in disease-onset classes, but employs common patients in the normal class. The results of combining normal patients with patients with DWI high signal, myocardial infarction, and stroke using pathological risk scores are shown in Fig. 2.

**Table 4:** Comparison of anomaly detection models (f-AnoGAN (WGAN-GP), f-AnoGAN (StyleGAN2), autoencoder) for pathological risk scores of 71 patients using Youden index thresholds

Model	ROC-AUC	Accuracy	Sensitivity	Specificity	F1-score
f-AnoGAN (WGAN-GP)	0.746	**0.817**	0.524	**0.940**	0.629
f-AnoGAN (StyleGAN2)	0.709	0.701	0.677	0.711	0.568
**Autoencoder**	**0.776**	0.775	**0.714**	0.800	**0.652**

The significance of bold best overall model for Pathlogical risk score, Classifier, and Model are in bold, and the highest value for each evaluation index is bold

### Risk factors for repeated cardiovascular events

**Fig. 3:**
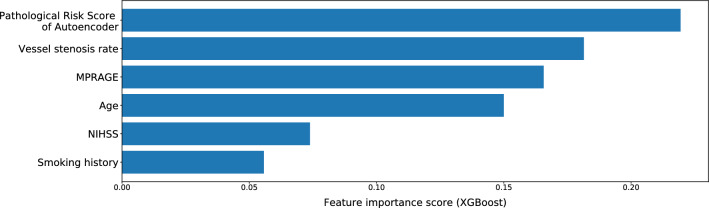
Variable importance scores (gain) for the top 6 predictors of cardiovascular disease development within one year after CEA in the XGBoost model (method #7)

Figure 3 shows the total gain of the XGBoost model. The feature importance score of the pathological risk score is higher than that of clinical data in the best-performing autoencoder + XGBoost model. This result indicated that the pathological risk score was more useful indicator to predict cardiovascular events, along with the high contribution of well-known factors, such as the vessel stenosis rate, MPRAGE to evaluate the vulnerable/unstable plaque, patient’s age, NIHSS, and smoking history.

### Visualization of abnormality on pathological image

**Fig. 4:**
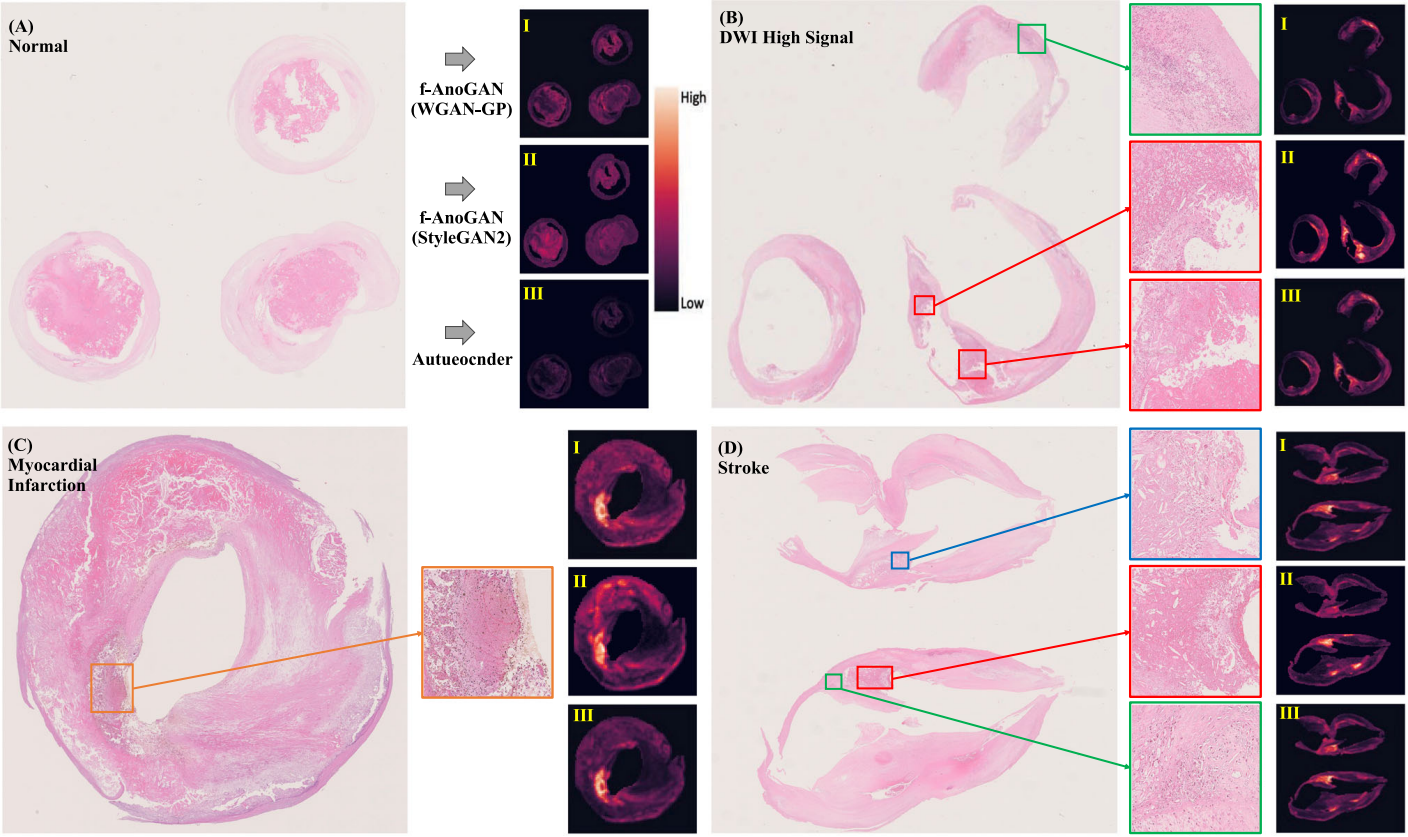
Pathological image and heat maps (from top, f-AnoGAN (WGAN-GP) (I), f-AnoGAN (StyleGAN2) (II), and autoencoder (III)) of normal patient (**A**), DWI high signal (**B**), myocardial infarction (**C**), and stroke (**D**) (red: plaque hemorrhage, orange: mural thrombus, green: lymphocyte infliction, blue: hemosiderin deposition)

Based on the anomaly score $\text{A}\left(x\right)$ for each patch image obtained in our experiment, we created heat maps to visualize which areas were abnormal on the pathological image. The higher the brightness of the image, the higher the anomaly score and the higher the risk of developing cardiovascular disease. Figure 4 shows the pathological image and the heatmap with f-AnoGAN and autoencoder. In addition, for 71 WSIs of 50 normal patients and 31 WSIs of 21 abnormal patients consisting of a validation set of anomaly detection model training, we asked the collaborating pathologist to delineate all pathological characteristics regions and annotate by its histopathology (e.g., plaque hemorrhage, mural thrombus, lymphocyte infliction, and hemosiderin deposition). As shown in Fig. 4A, the heat maps of normal patients are generally low in brightness, while the heat maps of abnormal patients (Fig. 4B, C, D) are high in the areas of plaque hemorrhage surrounded by red, mural thrombus surrounded by orange, lymphocytic infiltration surrounded by green, and hemosiderin deposition surrounded by blue, which are consistent with the findings of the pathologist, suggesting an association with pathological factors associated with the development of cardiovascular disease.

## Discussion

### Improved classification system by combining pathological images with clinical data

In this study, we aimed to improve the accuracy of cardiovascular events prediction after CEA by using pathological images and clinical data. Comparing the results of methods #3 and #7 in Table 3 and the autoencoder model in Table 4, ROC-AUC was 0.776 for pathological images alone and 0.701 for clinical data alone, while the combination improved to 0.819. The F1-score was also improved from 0.652 with pathological images alone and 0.640 with clinical data alone to 0.766 by the combination. These results show that combining pathological images with clinical data has the potential to improve the predictive power of classification systems. Pathological images provide important visual cues related to disease progression and capture the morphology of carotid plaques. Clinical data, on the other hand, provide broader background information, such as patient demographics, medical history, and other health factors. By combining pathological images with clinical data, our model can consider not only the visual signs of disease, but also individual patient characteristics, allowing for more comprehensive and accurate disease prediction.

### Improvement of pathological risk scores by anomaly detection on prediction accuracy

In the proposed method, an anomaly detection model is used to calculate a pathological risk score for each patient, which is then combined with clinical data to predict the onset of cardiovascular disease through a classification model. The results of prediction based on pathological risk scores alone (Table 4) showed that autoencoder performed the best, followed by f-AnoGAN (WGAN-GP) and f-AnoGAN (StyleGAN2). This result is also consistent with the ranking of performance in methods #3, #5, and #7 in Table 3. In addition, the highest importance of the pathological risk score from the feature importance in Fig. 3, proving the accuracy of the anomaly detection model for calculating the pathological risk score, may contribute to improving the accuracy of predicting the onset of cardiovascular disease when combined with clinical data.

As shown in Fig. 2, the F1-score within each disease was best for DWI, followed by myocardial infarction and stroke. This may be because myocardial infarction and stroke occurred within one year after surgery, whereas DWI high signal occurred immediately after surgery, making them easier to predict.

### Implications for future treatment and clinical practice

In this study, the heat map in Fig. 4 suggests that thrombus, lymphocytic infiltration, and hemosiderin deposition in pathological images, and vascular stenosis rate and MPRAGE value in clinical data may be important markers related to cardiovascular events. Although the number of subjects in this study was insufficient, validation with a large dataset could confirm the validity of these markers and models which can be used for early risk assessment and therapeutic intervention.

Furthermore, the proposed methods have the potential to reduce the burden on pathologists. Currently, pathologists are required to examine all areas of a WSI, which is inefficient given the current shortage of pathologists. However, the pathological risk score calculated by the proposed method allows the pathologist to focus on patients with high risk scores, which expects a more efficient diagnosis to be made.

## Conclusion

This paper proposed a predictive method for cardiovascular events within one year after CEA using pathological images and clinical data. The numerical evaluation showed that the autoencoder + XGBoost model achieved the highest F1-score (0.766) and ROC-AUC (0.819) using the pathological risk score and clinical data. This paper shows that pathological risk scores focusing on plaque hemorrhage, thrombus, lymphocytic infiltration, and hemosiderin deposition, along with clinical factors, are the indices that contribute most to the prediction of cardiovascular events. Furthermore, the proposed method has the potential to predict prognosis from thrombus pathology, etc., regardless of the CEA specimen. This is expected to identify common pathological features and biomarkers in different pathological images and to develop a new prognostic model using these features and biomarkers.

Although experiments produced promising results, it is limited by the small number of patients with event onset. The pathological risk score and pathological factors are reliable because the anomaly detection model was trained using 70 patients without events. However, the classification model was trained and verified on 50 patients without events and only 21 patients with events. It should be noted that the abnormal class includes patients with DWI high signal, which may be due to the surgical techniques used during CEA. Another limitation is that many recently proposed anomaly detection methods still need to be explored in our study. In the future, when more data are collected, we would like to consider experiments using the diffusion model.
